# Supplementary material for: Researcher population pyramids: Tracking demographic and gender trajectories across countries
Source: PNAS Nexus. 2026 Mar 10;5(3):pgag059. doi: 10.1093/pnasnexus/pgag059 (PMC12997530; doi:10.1093/pnasnexus/pgag059)
Supplement: pgag059_Supplementary_Data [file pgag059_supplementary_data.pdf]

Supplementary Information for: Researcher Population Pyramids: Tracking Demographic and Gender Trajectories Across Countries

Kazuki Nakajima and Takayuki Mizuno

S1 Gender Classification from Names

This section details the construction and evaluation of the Naive Bayes classifier used to infer gender from author names. We describe the data sources for our training, validation, and test sets, the classifier, and the results of our benchmark evaluations.

S1.1 Data Sources and Benchmark Set Construction

Our classifier was primarily trained and validated using data from Orbis, while WGND was used for additional tests of the model's generalizability. We detail the construction of benchmark datasets from both sources.

S1.1.1 Orbis Data

We used the April 2024 snapshot of Moody's Orbis database [1]. This database contains records for 69,020,795 executives, each providing their first name, binary gender, and a unique nationality. The nationalities in the dataset span 225 distinct ISO 3166-1 alpha-2 country codes. We extracted executives from the Orbis database, grouping them by their nationality.

We established specific data requirements for constructing training, validation, and test sets from Orbis data. For each country included in our benchmarks, we aimed to create balanced validation and test sets, each comprising 450 female and 450 male executives. This specific sample size was selected primarily due to the limited data availability for Tunisia in the Orbis database, which recorded only 939 female executives. We ensured that the training set for each gender was at least five times the size of the test set, necessitating a minimum of 2,250 individuals for training. Because our analysis has a particular focus on Arab countries, we accommodated countries with varying levels of data availability. This strategy involved a specific measure for data-scarce Arab nations: we supplemented their training data by incorporating names from other Arab countries that possessed more extensive datasets.

Based on these criteria, we categorized and selected the countries according to the following three conditions:

1. The country belongs to the Arab League (see Table S1) and has at least 3,150 female and 3,150 male executives. This threshold directly corresponds to our requirement of a 450-person test set, a 450-person validation set, and at least a 2,250-person training set per gender.

2. The country belongs to the Arab League, has at least 900 female and 900 male executives (sufficient for a test set and a validation set), but has fewer than 3,150 individuals in at least one gender group.

3. The country does not belong to the Arab League and has at least 3,150 female and 3,150 male executives.

This filtering process resulted in the identification of ten countries satisfying the first condition (i.e., Algeria, Bahrain, Egypt, Jordan, Kuwait, Lebanon, Morocco, Oman, Saudi Arabia, and the United Arab Emirates), four countries satisfying the second condition (i.e., Iraq, Somalia, Syria, and Tunisia), and 70 countries satisfying the third condition. We excluded the countries that did not satisfy any of these conditions from the benchmark analysis.

For the 80 countries satisfying the first or third condition, we stratified and randomly partitioned 450 male and 450 female executives each to the test set and validation set, with the remaining individuals forming the country-specific training set. For the four countries satisfying the second condition, we created the test and validation sets in the same manner. For their training set, however, we utilized a pooled Arab training set, aggregated from the training sets of all ten Arab countries that satisfied the first condition. Finally, to address potential gender imbalance within the training sets, we downsampled the majority gender group to ensure the ratio of the minority group to the majority group was at least 0.2.

S1.1.2 World Gender Name Dictionary Data

To test the generalizability of our classifier beyond the Orbis data, we used the World Gender Name Dictionary (WGND) [2, 3]. The WGND ver. 2.0 contains 5,016,972 entries, each including a first name, binary gender, country, and the frequency of that name-gender combination within the country. These frequencies are primarily drawn from census and population registration data, though some sources reflect multi-year aggregations or alternative datasets [2].

Among the 84 countries processed for the Orbis benchmark sets, 65 countries also possessed at least 500 unique names per gender in the WGND to enable sampling for validation and test sets. For each of these 65 countries, we constructed a validation set and a test set independently by sampling 10,000 names (5,000 female and 5,000 male) from the WGND data. We drew these samples using separate random number generators for each set and country. For both the validation and test sets, we performed the sampling with replacement weighted by the reported frequencies of each name-gender pair. This approach allows us to simulate a realistic population sample while ensuring a sufficient number of samples.

Overall, our benchmark construction process resulted in a total of 65 countries with complete benchmark sets across both Orbis and WGND datasets. The Orbis validation and test sets for each of these countries consist of 450 females and 450 males each. The WGND validation and test sets for each country consist of 5,000 females and 5,000 males each. Table S2 shows the number of executives in the training set constructed from the Orbis data for each country and gender.

S1.2 Naive Bayes Classifier

To prepare the data for our classifier, we first converted each first name into a feature vector. To ensure a consistent and comparable feature space across all countries, we tokenized every first name from the aggregated training corpus into character-level n-grams. We set the maximum n-gram length to the length of the longest name within this training corpus. We then transformed

these tokenized names into high-dimensional vectors using a Term Frequency-Inverse Document Frequency (TF-IDF) weighting scheme, where each component represents the TF-IDF weight of a specific character n-gram.

Using these feature vectors, we trained a separate gender classifier for each of the 65 countries. We chose the Naive Bayes algorithm, a probabilistic classification method based on Bayes' theorem, which operates under the assumption of conditional independence among features. Specifically, we employed the Complement Naive Bayes (CNB) variant, available in the 'scikit-learn' Python library [4, 5]. We selected CNB for its robustness in handling imbalanced datasets, as it estimates feature parameters by considering features from all classes except the one being evaluated. Furthermore, to counteract any remaining gender imbalance in the training data, we applied class-balanced sample weights during the training process.

The CNB classifier utilizes a smoothing hyperparameter, α, that addresses the zero-frequency problem and enhances the model's robustness against unseen data and class imbalance. We tuned a country-specific smoothing hyperparameter, α_c, of the CNB classifier for country c using its validation set constructed from the Orbis data. To this end, for each $\alpha_c \in \{0.001, 0.01, 0.1, 1, 10\}$, we evaluated the trained CNB classifier by calculating the Area Under the Receiver Operating Characteristic Curve (ROC AUC) on this validation set constructed from the Orbis data for each country. We used the tuned α_c value that yielded the highest ROC AUC for each country (see Table S3).

To ensure the reliability of gender assignments, among the 65 countries with both Orbis and WGND validation sets available, we retained the 61 where the CNB classifier achieved an ROC AUC of at least 0.8 on both validation sets. This criterion led to the exclusion of four countries (Bangladesh, China, India, and South Korea) due to their lower ROC AUC values on the WGND validation set.

S1.3 Country-Specific Thresholding for Gender Assignment

To ensure the high reliability of our gender assignments, we applied a country-specific confidence threshold, θ_c, to probability estimates produced by our CNB classifier. We assign gender g to a name associated with country c if and only if an estimate of the posterior probability of that name's gender g is θ_c or larger; otherwise, we do not assign it.

For each of the 61 countries, we determined these thresholds based on the classifiers' performance on both Orbis and WGND validation sets. We defined a target F1 score of 0.9 as a practical standard for high-confidence assignments. The F1 score was computed only on the subset of instances in the validation set meeting the confidence threshold (i.e., $p_g \geq \theta$), with the male class treated as positive.

To find θ_c, we examined a range of candidate thresholds from 0.9 to 0.9999. For each country, we determined the smallest threshold that achieved an F1 score of at least 0.9 on the Orbis validation set (θ_c^{Orbis}) and, separately, on the WGND validation set (θ_c^{WGND}). If the F1 score did not reach 0.9 even at a threshold of 0.9999, we consistently set the respective threshold (θ_c^{Orbis} or θ_c^{WGND}) to 0.9999 for that country. The final confidence threshold, θ_c, was set to the maximum of these two values ($\theta_c = \max(\theta_c^{\text{Orbis}}, \theta_c^{\text{WGND}})$). The resulting thresholds, $\{\theta_c\}_c$, along with the tuned smoothing hyperparameters, $\{\alpha_c\}_c$, for all 61 countries are listed in Table S3.

For comparison, we also performed country-specific tuning of its confidence threshold, θ_c, for a name-based gender classifier that uses cultural consensus theory (the CCT classifier [6]). We followed the same procedure used for tuning θ_c of our CNB classifier for this purpose. The resulting thresholds, $\{\theta_c\}_c$ for all 61 countries are listed in Table S4.

S1.4 Benchmark Evaluation

We evaluated our trained CNB classifier on both the Orbis and WGND test sets for each country by calculating the ROC AUC and F1 score. We also computed the gender assignment rate (GAR)—the fraction of gender-assigned test samples—separately for female and male samples (GAR_f and GAR_m). For comparison, we computed the same set of metrics for the CCT classifier. For all these metrics, higher values indicate better performance.

Table S5 presents the benchmark results for both classifiers across all 61 countries in the Orbis test sets. As expected, the CNB classifier generally outperforms or matches the CCT classifier, given that the CNB model is directly trained on Orbis data. Across these 61 countries, the CNB classifier achieved (mean ± standard deviation) ROC AUCs of 0.979 ± 0.034, F1 scores of 0.973 ± 0.026, and gender assignment rates of 0.896 ± 0.131 for female and 0.907 ± 0.117 for male test samples. In comparison, the CCT classifier achieved ROC AUCs of 0.960 ± 0.048, F1 scores of 0.970 ± 0.029, with corresponding gender assignment rates of 0.827 ± 0.137 for female and 0.864 ± 0.112 for male test samples.

Table S6 presents the benchmark results for both classifiers across all 61 countries in the WGND test sets. In contrast to Orbis, the CNB classifier performs comparably to or worse than the CCT classifier on the WGND test sets. Across these 61 countries, the CNB classifier achieved (mean ± standard deviation) ROC AUCs of 0.938 ± 0.049, F1 scores of 0.943 ± 0.036, and gender assignment rates of 0.748 ± 0.198 for female and 0.729 ± 0.211 for male test samples. In comparison, the CCT classifier achieved ROC AUCs of 0.987 ± 0.020, F1 scores of 0.992 ± 0.013, with corresponding gender assignment rates of 0.845 ± 0.121 for female and 0.839 ± 0.104 for male test samples.

The two classifiers exhibit complementary strengths tied to the nature of the underlying datasets. The CCT classifier demonstrates superior generalization performance on the WGND benchmark, which is largely derived from general population data. In contrast, our CNB classifier, trained on the Orbis database of international executives, often outperforms the CCT classifier on the Orbis test set. This observed difference also illustrates the inherent difficulty of inferring gender from names across countries and emphasizes the importance of cross-dataset evaluation.

For the main analysis, we opted to use the CNB classifier. This decision was motivated by the specific nature of our target population: academic researchers. Researchers, much like the executives in the Orbis database, may constitute a highly mobile international population, a characteristic often termed "academic mobility" [7]. We reasoned that the demographic and naming patterns of this group might be more accurately captured by the Orbis data than by the more static, country-specific general population data represented by WGND. Therefore, we judged the CNB classifier to be a suitable choice for this specific analytical context.

S2 Number of authors by gender and country

Table S7 lists the number of gender-assigned authors for each of the 61 countries that passed our initial accuracy benchmarks. For the main analysis, however, we applied an additional filtering step to ensure that each country had a sufficiently large population for robust demographic analysis. Specifically, we retained only those countries with at least 1,000 authors for each gender. This criterion led to the exclusion of three countries: Lesotho, Papua New Guinea, and Somalia. The final analysis, therefore, focuses on the remaining 58 countries.

S3 Analysis of Trends in Researcher Populations and Gender Balance

S3.1 Researcher Populations

This section details the methodology for comparing long-term trends in researcher population growth between the nine Arab countries and the ten reference countries.

For each country c, we began by computing $N_c(t)$, the total number of gender-assigned authors affiliated with country c who published at least one paper in year $t \in [2000, 2023]$. To analyze the long-term growth, we then transformed this population count into a log-linear trajectory, $y_c(t) = \log N_c(t)$, where $N_c(t) > 0$ for all c and t considered. For each country, we fit the following linear regression model to the log-transformed annual author counts:

$$y_c(t) = \beta_{c,0} + \beta_c t + \varepsilon_c(t), \tag{S1}$$

where $\beta_{c,0}$ is the intercept term, β_c is the estimated slope representing the annual growth rate, and $\varepsilon_c(t)$ is the residual term.

We obtained the estimated slopes $\{\beta_c\}_c$ for each of the nine Arab countries and the ten reference countries. To account for potential autocorrelation in the time-series data, we computed standard errors (SEs) and p-values using a Newey-West estimator. Table S8 reports the estimated slope β_c, its corresponding SE and p-value, and the coefficient of determination R^2 for each country.

S3.2 Gender Balance

This section details the methodology for examining long-term trends in gender balance, specifically focusing on the proportion of female authors for each country over the period 2000–2023.

For each country c, we computed $p_c(t)$, the proportion of female authors among all active authors in year t. We then fit the following linear regression model to the annual time series $\{p_c(t)\}$ for each country to estimate the trend in female author representation:

$$p_c(t) = \gamma_{c,0} + \gamma_c t + \delta_c(t), \tag{S2}$$

where $\gamma_{c,0}$ is the intercept term, γ_c is the estimated slope representing the average annual change in the proportion of female authors, and $\delta_c(t)$ is the residual term.

We obtained the estimated slopes $\{\gamma_c\}_c$ for each of the nine Arab countries and the ten reference countries. To account for potential autocorrelation in the time-series data, we computed standard errors (SEs) and p-values using a Newey-West estimator. Table S9 reports the estimated slope γ_c, its corresponding SE and p-value, and the coefficient of determination R^2 for each country.

S4 Inter-publication Interval Threshold by Country and Gender

Table S10 shows the inter-publication interval thresholds, $\Delta_{\text{IPI},c,g}$, by country c and gender g when the survival probability threshold is set to 1%, 2%, and 5%.

S5 Analyses of Authors' Career Metrics

We computed several career metrics for all authors active in 2023. In addition to our primary metric, cumulative productivity, we calculated the following for each author:

- **Total productivity:** The total number of publications by the end of 2023.
- **Publishing-career length:** The duration in years from their first publication to the end of 2023.
- **Publication gap experience:** A binary indicator, true if the author has ever experienced an inter-publication interval longer than their corresponding $\Delta_{\mathrm{IPI},c,g}$ threshold.
- **Returning author status:** A binary indicator, true if the author was inactive in 2022 but became active in 2023, and had published at least one paper before 2023.

We first calculated the Spearman's rank correlation between cumulative productivity, total productivity, and publishing-career length for each country and gender (see Table S11). The Spearman's rank correlation between cumulative productivity and total productivity was, as expected, very high, given that both are based on publication counts. However, the correlation was not perfect, which reflects that cumulative productivity is sensitive to the continuity of publication activity. In contrast, the correlation between cumulative productivity and publishing-career length was only moderate. This weaker correlation suggests that cumulative productivity is not a simple proxy for the length of publishing-career but rather captures an author's recent publication momentum.

We further analyzed two metrics: the proportion of all active authors with a publication gap experience (see Table S12), and the proportion of returning authors within newly active authors (see Table S13). First, we observed a contrast between country groups. For both metrics, the proportions were substantially higher in the reference countries than in the Arab countries. On average, the proportion of authors with a publication gap experience was substantially higher in the reference countries (15.6% for female; 24.4% for male) than in the Arab countries (6.6% for female; 12.1% for male). Similarly, on average, the proportion of returning authors was substantially higher in reference countries (15.7% for female; 25.4% for male) than in Arab countries (5.7% for female; 9.5% for male). Second, we found a consistent gender difference across both metrics. In 56 out of 58 countries, a larger proportion of male authors had a publication gap experience compared to their female counterparts. Likewise, in 57 out of 58 countries, the proportion of returning authors was higher for men than for women. This counter-intuitive result is considered a consequence of our gender-specific inter-publication interval thresholds, which are empirically derived and are longer for women in most countries (48 out of 58 countries; see Table S10).

S6 Researcher Inflow and Gender Gap in Cumulative Productivity by Country in 2023

Table S14 shows the researcher flow and the gender gap in cumulative productivity for each country in 2023.

S7 Compound Annual Growth Rate of the Number of Active Authors

We define the compound annual growth rate (CAGR) of the number of active authors from year t_1 to year t_2 as $(n_2/n_1)^{1/(t_2-t_1)} - 1$, where n_1 and n_2 denote the number of active authors in years t_1 and t_2, respectively.

S8 Analysis Across Research Domains

S8.1 Methods

We additionally conducted our analysis across the four high-level research domains defined by OpenAlex: "Health Sciences", "Life Sciences", "Physical Sciences", and "Social Sciences". Following the methodology used for country of affiliation (Section 4.1), we assigned research domains to each author based on the frequency of research domains associated with their publications. Specifically, we identified the research domain(s) that appeared most frequently across an author's entire publication record. We retained only those authors whose research domain was uniquely determined (i.e., those with a single primary domain and no ties in frequency). For each combination of target year, country, and research domain, we constructed researcher population pyramids using the same criteria and methodology as those used to compute the researcher population pyramids shown in Fig. 2 in the main text.

S8.2 Results

Figures S1–S4 present the researcher population pyramids for Egypt, Tunisia, Japan, and the United States across the four research domains for the years 2010, 2023, and 2050 (Health Sciences in Fig. S1, Life Sciences in Fig. S2, Physical Sciences in Fig. S3, and Social Sciences in Fig. S4). Our analysis reveals two key findings. First, the vertical reach of the pyramids varies across domains, reflecting distinct disciplinary publication norms; for example, researchers in the Health Sciences exhibit higher maximum levels of cumulative productivity, while those in the Social Sciences exhibit lower overall publication volumes. Second, despite these differences in vertical scale, the qualitative characteristics of the three research systems remain largely consistent within each country across all four domains. Egypt and Tunisia consistently exhibit the "Emerging" pattern; the United States exhibits the "Mature" pattern; and Japan exhibits the "Rigid" pattern in every domain analyzed. These results indicate that while disciplinary norms influence the absolute height (cumulative productivity levels) of the pyramids, our framework captures the demographic trajectories of national research ecosystems.

References

[1] Moody's Corporation. Orbis. `https://www.moodys.com/web/en/us/capabilities/company-reference-data/orbis.html`. Accessed May, 2025.

[2] World Intellectual Property Organization. Expanding the World Gender-Name Dictionary: WGND 2.0. `https://tind.wipo.int/record/43980?v=pdf`. Accessed May, 2025.

[3] World Intellectual Property Organization. World Gender Name Dictionary 2.0 Dataset. https://tind.wipo.int/record/49408?ln=en&v=zip. Accessed May, 2025.

[4] Jason D Rennie, Lawrence Shih, Jaime Teevan, and David R Karger. Tackling the poor assumptions of naive bayes text classifiers. In *Proceedings of the 20th international conference on machine learning (ICML-03)*, pages 616–623, 2003.

[5] F. Pedregosa, G. Varoquaux, A. Gramfort, V. Michel, B. Thirion, O. Grisel, M. Blondel, P. Prettenhofer, R. Weiss, V. Dubourg, J. Vanderplas, A. Passos, D. Cournapeau, M. Brucher, M. Perrot, and E. Duchesnay. Scikit-learn: Machine learning in Python. *Journal of Machine Learning Research*, 12:2825–2830, 2011.

[6] Ian Van Buskirk, Aaron Clauset, and Daniel B. Larremore. An open-source cultural consensus approach to name-based gender classification. *Proceedings of the International AAAI Conference on Web and Social Media*, 17:866–877, 2023.

[7] Louise Ackers. Internationalisation, mobility and metrics: A new form of indirect discrimination? *Minerva*, 46:411–435, 2008.

[8] League of arab states. https://unterm.un.org/unterm2/en/view/UNHQ/C17E5C77C7F88D3D852569FA000082D2. Accessed May, 2025.

Table S1: Member States of the Arab League [8].

Country
Algeria
Bahrain
Comoros
Djibouti
Egypt
Iraq
Jordan
Kuwait
Lebanon
Libya
Mauritania
Morocco
Oman
Palestine, State of
Qatar
Saudi Arabia
Somalia
Sudan
Syrian Arab Republic
Tunisia
United Arab Emirates
Yemen

Table S2: Number of samples used in the training set for each country.

Country	Female	Male
Albania	4,560	22,800
Algeria	27,413	137,065
Australia	45,789	104,429
Austria	10,746	36,555
Bahrain	35,066	45,000
Bangladesh	5,515	22,171
Belarus	4,385	12,505
Belgium	8,741	32,846
Bosnia and Herzegovina	2,741	8,511
Bulgaria	609,226	822,719
Canada	18,289	62,179
China	90,973	222,418
Cyprus	2,339	9,200
Czechia	153,267	304,260
Denmark	9,701	30,685
Egypt	18,316	91,580
Estonia	8,272	15,792
France	61,820	170,251
Germany	82,796	269,500
Ghana	4,637	12,014
Iceland	22,307	46,856
India	94,220	305,744
Iran, Islamic Republic of	7,050	35,250
Iraq	273,599	1,041,777
Ireland	70,759	149,920
Israel	4,357	21,785
Italy	2,508,071	5,348,406
Jamaica	4,754	7,588
Japan	9,200	46,000
Jordan	71,967	359,835
Kenya	16,919	59,825
Korea, Republic of	4,781	23,905
Kuwait	14,005	49,845
Lebanon	55,521	130,120
Lesotho	8,961	14,983
Lithuania	11,419	26,476
Moldova, Republic of	20,717	39,152
Montenegro	5,990	13,186
Morocco	3,075	15,375
Netherlands	21,159	77,841
New Zealand	314,854	517,150
Nigeria	26,410	55,444
Norway	425,903	694,519

Country	Female	Male
Oman	33,653	140,042
Papua New Guinea	35,812	114,524
Philippines	61,500	40,699
Poland	55,843	133,137
Portugal	18,029	44,429
Romania	611,956	948,609
Russian Federation	6,808,203	10,064,454
Saudi Arabia	5,111	25,555
Serbia	174,091	330,888
Somalia	273,599	1,041,777
South Africa	3,060,324	3,829,095
Spain	23,016	60,053
Sri Lanka	4,841	13,854
Sweden	24,125	62,170
Switzerland	376,575	746,801
Syrian Arab Republic	273,599	1,041,777
Tunisia	273,599	1,041,777
Turkey	17,420	87,100
United Arab Emirates	9,472	47,360
United Kingdom	4,735,568	7,910,437
United States	70,070	259,774
Zimbabwe	8,837	11,148

Table S3: Country-specific tuned hyperparameters of our CNB classifier.

Country	α_c	θ_c
Albania	0.100	0.9000
Algeria	1.000	0.9000
Australia	0.010	0.9000
Austria	0.001	0.9700
Bahrain	0.010	0.9930
Belarus	0.001	0.9992
Belgium	0.100	0.9000
Bosnia and Herzegovina	0.100	0.9000
Bulgaria	0.001	0.9000
Canada	0.100	0.9000
Cyprus	0.010	0.9900
Czechia	10.000	0.9000
Denmark	0.100	0.9000
Egypt	0.100	0.9000
Estonia	0.100	0.9000
France	0.100	0.9000
Germany	1.000	0.9000
Ghana	0.100	0.9000
Iceland	0.010	0.9000
Iran, Islamic Republic of	0.100	0.9400
Iraq	0.100	0.9998
Ireland	0.100	0.9000
Israel	0.100	0.9000
Italy	0.001	0.9000
Jamaica	0.100	0.9000
Japan	0.100	0.9000
Jordan	0.100	0.9300
Kenya	0.100	0.9400
Kuwait	0.010	0.9800
Lebanon	0.010	0.9800
Lesotho	0.100	0.9000
Lithuania	0.001	0.9000
Moldova, Republic of	0.001	0.9900
Montenegro	0.100	0.9000
Morocco	1.000	0.9000
Netherlands	0.100	0.9000
New Zealand	0.100	0.9000
Nigeria	0.100	0.9000
Norway	0.100	0.9000
Oman	0.010	0.9960
Papua New Guinea	0.100	0.9000
Philippines	1.000	0.9000
Poland	0.001	0.9940

Country	α_c	θ_c
Portugal	0.100	0.9000
Romania	10.000	0.9000
Russian Federation	0.001	0.9400
Saudi Arabia	0.100	0.9500
Serbia	0.001	0.9600
Somalia	0.010	0.9999
South Africa	0.010	0.9000
Spain	0.100	0.9000
Sri Lanka	1.000	0.9000
Sweden	0.100	0.9000
Switzerland	0.010	0.9000
Syrian Arab Republic	0.100	0.9300
Tunisia	0.100	0.9000
Türkiye	0.100	0.9000
United Arab Emirates	0.001	0.9970
United Kingdom	1.000	0.9000
United States	0.100	0.9000
Zimbabwe	0.010	0.9000

Table S4: Country-specific tuned hyperparameters of the CCT classifier.

Country	θ_c
Albania	0.9000
Algeria	0.9000
Australia	0.9000
Austria	0.9000
Bahrain	0.9000
Belarus	0.9000
Belgium	0.9000
Bosnia and Herzegovina	0.9000
Bulgaria	0.9000
Canada	0.9000
Cyprus	0.9000
Czechia	0.9000
Denmark	0.9000
Egypt	0.9000
Estonia	0.9000
France	0.9000
Germany	0.9000
Ghana	0.9000
Iceland	0.9000
Iran, Islamic Republic of	0.9000
Iraq	0.9000
Ireland	0.9000
Israel	0.9000
Italy	0.9000
Jamaica	0.9000
Japan	0.9000
Jordan	0.9000
Kenya	0.9000
Kuwait	0.9000
Lebanon	0.9000
Lesotho	0.9999
Lithuania	0.9000
Moldova, Republic of	0.9000
Montenegro	0.9000
Morocco	0.9000
Netherlands	0.9000
New Zealand	0.9000
Nigeria	0.9000
Norway	0.9000
Oman	0.9000
Papua New Guinea	0.9000
Philippines	0.9000
Poland	0.9000

Country	θ_c
Portugal	0.9000
Romania	0.9000
Russian Federation	0.9000
Saudi Arabia	0.9300
Serbia	0.9000
Somalia	0.9000
South Africa	0.9000
Spain	0.9000
Sri Lanka	0.9000
Sweden	0.9000
Switzerland	0.9000
Syrian Arab Republic	0.9000
Tunisia	0.9000
Türkiye	0.9000
United Arab Emirates	0.9000
United Kingdom	0.9000
United States	0.9000
Zimbabwe	0.9000

Table S5: Benchmark results on the Orbis test sets for the CNB and CCT classifiers. Columns AUC and F1 represent the ROC AUC and F1 score, respectively. GAR_f and GAR_m represent the gender assignment rates for the female and male test samples, respectively.

	CNB				CCT			
Country	AUC	F1	GAR_f	GAR_m	AUC	F1	GAR_f	GAR_m
Albania	0.984	0.963	0.902	0.911	0.950	0.963	0.853	0.869
Algeria	0.965	0.951	0.880	0.931	0.945	0.953	0.847	0.911
Australia	0.995	0.978	0.964	0.947	0.992	0.994	0.891	0.931
Austria	0.996	0.984	0.962	0.978	0.995	0.996	0.927	0.967
Bahrain	0.998	0.998	0.960	0.956	0.969	0.969	0.824	0.891
Belarus	0.999	0.999	0.951	0.980	0.997	0.998	0.989	0.967
Belgium	0.982	0.971	0.887	0.873	0.979	0.985	0.896	0.864
Bosnia and Herzegovina	0.986	0.974	0.889	0.927	0.979	0.978	0.896	0.920
Bulgaria	0.998	0.993	0.996	0.984	0.987	0.991	0.933	0.798
Canada	0.987	0.973	0.893	0.918	0.984	0.986	0.851	0.918
Cyprus	0.976	0.974	0.749	0.889	0.971	0.970	0.847	0.931
Czechia	0.999	0.995	0.967	0.951	0.999	0.999	0.953	0.909
Denmark	0.989	0.977	0.884	0.931	0.987	0.987	0.864	0.893
Egypt	0.988	0.968	0.924	0.953	0.945	0.944	0.733	0.849
Estonia	0.998	0.994	0.956	0.969	0.991	0.992	0.847	0.851
France	0.995	0.986	0.960	0.916	0.991	0.995	0.902	0.858
Germany	0.995	0.991	0.944	0.933	0.995	0.995	0.931	0.940
Ghana	0.994	0.982	0.938	0.920	0.975	0.986	0.876	0.896
Iceland	0.999	0.997	0.984	0.987	0.988	0.990	0.902	0.918
Iran, Islamic Republic of	0.981	0.974	0.860	0.844	0.933	0.941	0.769	0.856
Iraq	0.841	0.938	0.347	0.364	0.906	0.927	0.618	0.780
Ireland	0.999	0.997	0.958	0.971	0.998	0.998	0.918	0.969
Israel	0.965	0.953	0.822	0.889	0.941	0.952	0.811	0.820
Italy	0.999	0.999	0.998	0.999	0.999	0.999	0.991	0.860
Jamaica	0.978	0.969	0.838	0.833	0.964	0.972	0.762	0.816
Japan	0.981	0.956	0.904	0.964	0.967	0.970	0.733	0.882
Jordan	0.995	0.986	0.971	0.958	0.951	0.954	0.736	0.867
Kenya	0.914	0.895	0.918	0.922	0.907	0.896	0.938	0.929
Kuwait	0.985	0.968	0.898	0.927	0.928	0.942	0.631	0.876
Lebanon	0.997	0.992	0.942	0.976	0.926	0.931	0.802	0.929
Lesotho	0.975	0.965	0.887	0.847	0.687	0.858	0.147	0.213
Lithuania	0.994	0.991	0.998	0.993	0.994	0.994	0.920	0.971
Moldova, Republic of	0.993	0.986	0.987	0.960	0.985	0.986	0.969	0.913
Montenegro	0.983	0.955	0.916	0.920	0.955	0.959	0.864	0.889
Morocco	0.989	0.985	0.898	0.893	0.973	0.976	0.911	0.913
Netherlands	0.981	0.973	0.864	0.931	0.980	0.984	0.864	0.896
New Zealand	0.998	0.992	0.962	0.942	0.989	0.991	0.898	0.893
Nigeria	0.954	0.923	0.862	0.869	0.933	0.952	0.604	0.742
Norway	0.991	0.980	0.980	0.987	0.984	0.986	0.893	0.887
Oman	0.999	0.997	0.958	0.980	0.908	0.918	0.742	0.851

	CNB				CCT			
Country	AUC	F1	GAR_f	GAR_m	AUC	F1	GAR_f	GAR_m
Papua New Guinea	0.983	0.969	0.887	0.907	0.946	0.970	0.764	0.784
Philippines	0.981	0.978	0.873	0.849	0.958	0.968	0.784	0.822
Poland	0.992	0.990	0.982	0.973	0.991	0.993	0.973	0.929
Portugal	0.996	0.992	0.933	0.913	0.992	0.994	0.924	0.924
Romania	0.980	0.967	0.922	0.920	0.962	0.973	0.851	0.787
Russian Federation	0.999	0.996	0.993	0.998	0.998	0.999	0.964	0.929
Saudi Arabia	0.981	0.975	0.858	0.933	0.878	0.901	0.684	0.849
Serbia	0.997	0.994	0.967	0.987	0.984	0.979	0.949	0.938
Somalia	0.824	0.883	0.280	0.480	0.964	0.971	0.749	0.867
South Africa	0.979	0.962	0.884	0.858	0.919	0.966	0.624	0.671
Spain	0.990	0.978	0.944	0.940	0.986	0.984	0.920	0.924
Sri Lanka	0.884	0.927	0.500	0.473	0.858	0.930	0.562	0.582
Sweden	0.996	0.988	0.922	0.949	0.988	0.992	0.933	0.909
Switzerland	0.999	0.992	0.989	0.991	0.999	0.999	0.962	0.933
Syrian Arab Republic	0.917	0.888	0.822	0.840	0.943	0.954	0.653	0.853
Tunisia	0.965	0.953	0.898	0.844	0.964	0.966	0.807	0.871
Türkiye	0.990	0.975	0.924	0.936	0.972	0.977	0.811	0.898
United Arab Emirates	0.995	0.991	0.933	0.969	0.942	0.950	0.742	0.902
United Kingdom	0.998	0.991	0.967	0.964	0.996	0.998	0.876	0.918
United States	0.987	0.968	0.942	0.944	0.978	0.984	0.849	0.933
Zimbabwe	0.970	0.952	0.891	0.862	0.941	0.972	0.771	0.658

Table S6: Benchmark results on the WGND test sets for the CNB and CCT classifiers. Columns AUC and F1 represent the ROC AUC and F1 score, respectively. GAR_f and GAR_m represent the gender assignment rates for the female and male test samples, respectively.

	CNB				CCT			
Country	AUC	F1	GAR_f	GAR_m	AUC	F1	GAR_f	GAR_m
Albania	0.934	0.918	0.781	0.842	0.997	0.998	0.916	0.855
Algeria	0.871	0.938	0.468	0.359	0.988	0.996	0.796	0.832
Australia	0.993	0.979	0.956	0.959	0.996	0.996	0.893	0.928
Austria	0.915	0.899	0.797	0.812	0.992	0.993	0.908	0.881
Bahrain	0.908	0.913	0.747	0.555	0.990	0.996	0.809	0.843
Belarus	0.859	0.907	0.439	0.414	0.902	0.936	0.708	0.648
Belgium	0.991	0.984	0.894	0.885	0.998	0.998	0.920	0.893
Bosnia and Herzegovina	0.962	0.954	0.762	0.781	0.986	0.991	0.904	0.882
Bulgaria	0.999	0.999	0.999	0.999	0.999	0.999	0.999	0.820
Canada	0.993	0.981	0.948	0.939	0.998	0.998	0.885	0.934
Cyprus	0.824	0.906	0.320	0.267	0.992	0.991	0.917	0.856
Czechia	0.959	0.965	0.768	0.670	0.970	0.975	0.847	0.783
Denmark	0.999	0.999	0.997	0.956	0.999	0.999	0.915	0.956
Egypt	0.895	0.904	0.649	0.601	0.988	0.996	0.814	0.834
Estonia	0.982	0.970	0.872	0.917	0.996	0.999	0.823	0.874
France	0.999	0.997	0.987	0.961	0.999	0.999	0.936	0.855
Germany	0.933	0.937	0.772	0.667	0.993	0.993	0.894	0.883
Ghana	0.906	0.923	0.673	0.590	0.992	0.989	0.914	0.854
Iceland	0.993	0.975	0.922	0.966	0.983	0.993	0.737	0.815
Iran, Islamic Republic of	0.857	0.892	0.526	0.516	0.996	0.998	0.856	0.893
Iraq	0.944	0.977	0.508	0.342	0.988	0.995	0.808	0.834
Ireland	0.999	0.993	0.983	0.976	0.999	0.999	0.930	0.958
Israel	0.937	0.933	0.734	0.759	0.988	0.991	0.852	0.800
Italy	0.995	0.991	0.985	0.988	0.999	0.999	0.946	0.910
Jamaica	0.909	0.929	0.608	0.576	0.992	0.990	0.907	0.852
Japan	0.928	0.908	0.759	0.811	0.945	0.970	0.625	0.726
Jordan	0.901	0.896	0.737	0.647	0.990	0.997	0.809	0.831
Kenya	0.886	0.906	0.516	0.633	0.992	0.991	0.910	0.847
Kuwait	0.888	0.901	0.655	0.556	0.990	0.996	0.808	0.836
Lebanon	0.897	0.906	0.693	0.597	0.990	0.996	0.807	0.838
Lesotho	0.846	0.908	0.375	0.474	0.992	0.994	0.089	0.259
Lithuania	0.953	0.927	0.909	0.942	0.982	0.991	0.837	0.829
Moldova, Republic of	0.871	0.908	0.559	0.524	0.896	0.933	0.689	0.638
Montenegro	0.888	0.932	0.439	0.526	0.993	0.994	0.912	0.888
Morocco	0.863	0.948	0.353	0.284	0.989	0.997	0.795	0.829
Netherlands	0.971	0.968	0.826	0.787	0.995	0.994	0.837	0.839
New Zealand	0.999	0.988	0.971	0.987	0.999	0.999	0.899	0.950
Nigeria	0.906	0.922	0.629	0.687	0.991	0.991	0.910	0.855
Norway	0.999	0.993	0.954	0.971	0.999	0.999	0.879	0.929
Oman	0.876	0.902	0.593	0.495	0.988	0.996	0.812	0.825

Country	CNB				CCT			
	AUC	F1	GAR_f	GAR_m	AUC	F1	GAR_f	GAR_m
Papua New Guinea	0.939	0.918	0.839	0.824	0.992	0.990	0.911	0.854
Philippines	0.999	0.999	0.912	0.979	0.996	0.990	0.919	0.999
Poland	0.910	0.906	0.688	0.738	0.966	0.989	0.811	0.660
Portugal	0.974	0.961	0.864	0.862	0.999	0.999	0.956	0.961
Romania	0.931	0.939	0.591	0.566	0.987	0.987	0.906	0.830
Russian Federation	0.934	0.895	0.864	0.872	0.923	0.958	0.722	0.683
Saudi Arabia	0.888	0.903	0.645	0.488	0.987	0.996	0.768	0.799
Serbia	0.923	0.905	0.727	0.791	0.989	0.989	0.914	0.867
Somalia	0.945	0.975	0.551	0.400	0.989	0.996	0.803	0.837
South Africa	0.978	0.954	0.913	0.897	0.991	0.991	0.896	0.859
Spain	0.997	0.992	0.950	0.946	0.992	0.999	0.844	0.857
Sri Lanka	0.909	0.966	0.201	0.406	0.989	0.995	0.808	0.744
Sweden	0.998	0.993	0.946	0.953	0.999	0.999	0.920	0.918
Switzerland	0.997	0.988	0.955	0.964	0.999	0.999	0.943	0.910
Syrian Arab Republic	0.943	0.918	0.874	0.713	0.989	0.996	0.803	0.835
Tunisia	0.943	0.913	0.889	0.747	0.988	0.996	0.803	0.834
Türkiye	0.983	0.970	0.887	0.889	0.994	0.994	0.835	0.853
United Arab Emirates	0.878	0.906	0.603	0.544	0.990	0.997	0.816	0.832
United Kingdom	0.995	0.984	0.951	0.930	0.998	0.998	0.889	0.882
United States	0.989	0.972	0.952	0.932	0.995	0.993	0.887	0.928
Zimbabwe	0.923	0.906	0.789	0.780	0.992	0.990	0.912	0.852

Table S7: Number of authors by gender, proportion of female authors, and gender assignment rate for each country. N: total number of authors with an assigned country. n_{female}: number of female authors. n_{male}: number of male authors. p_{female}: proportion of female authors among gender-assigned authors ($n_{\text{female}}/(n_{\text{female}} + n_{\text{male}})$). GAR: gender assignment rate ($(n_{\text{female}} + n_{\text{male}})/N$).

Country	N	n_{female}	n_{male}	p_{female} (%)	GAR (%)
Albania	8,101	3,636	2,484	59.4%	75.5%
Algeria	82,032	24,186	28,860	45.6%	64.7%
Australia	449,116	157,027	193,939	44.7%	78.1%
Austria	140,857	43,527	64,650	40.2%	76.8%
Bahrain	6,383	2,377	2,163	52.4%	71.1%
Belarus	32,530	3,665	4,458	45.1%	25.0%
Belgium	193,359	56,615	75,125	43.0%	68.1%
Bosnia and Herzegovina	15,878	6,959	5,584	55.5%	79.0%
Bulgaria	46,690	17,182	20,643	45.4%	81.0%
Canada	709,352	232,752	266,578	46.6%	70.4%
Cyprus	13,567	3,162	4,876	39.3%	59.2%
Czechia	138,070	40,978	55,632	42.4%	70.0%
Denmark	137,867	47,737	54,154	46.9%	73.9%
Egypt	273,642	97,512	110,256	46.9%	75.9%
Estonia	18,136	6,826	6,224	52.3%	72.0%
France	975,619	280,487	429,656	39.5%	72.8%
Germany	1,362,026	342,417	551,829	38.3%	65.7%
Ghana	35,647	7,631	19,671	28.0%	76.6%
Iceland	7,445	2,977	3,382	46.8%	85.4%
Iran, Islamic Republic of	580,434	208,756	246,589	45.8%	78.4%
Iraq	115,842	25,416	27,660	47.9%	45.8%
Ireland	74,655	26,894	28,408	48.6%	74.1%
Israel	165,123	51,135	69,100	42.5%	72.8%
Italy	671,119	239,535	294,198	44.9%	79.5%
Jamaica	3,938	1,194	995	54.5%	55.6%
Japan	1,426,766	268,393	849,383	24.0%	78.3%
Jordan	41,137	13,926	19,738	41.4%	81.8%
Kenya	51,264	12,196	22,554	35.1%	67.8%
Kuwait	17,140	4,271	6,733	38.8%	64.2%
Lebanon	21,983	9,538	7,541	55.8%	77.7%
Lesotho	826	219	262	45.5%	58.2%
Lithuania	32,502	16,170	12,388	56.6%	87.9%
Moldova, Republic of	9,729	3,510	3,164	52.6%	68.6%
Montenegro	2,679	1,148	942	54.9%	78.0%
Morocco	77,995	17,994	26,155	40.8%	56.6%
Netherlands	394,621	107,770	160,882	40.1%	68.1%
New Zealand	75,098	25,651	31,850	44.6%	76.6%
Nigeria	261,895	47,770	106,339	31.0%	58.8%
Norway	121,078	41,343	52,325	44.1%	77.4%
Oman	15,633	4,635	4,749	49.4%	60.0%

Country	N	n_{female}	n_{male}	p_{female} (%)	GAR (%)
Papua New Guinea	2,182	525	1,009	34.2%	70.3%
Philippines	79,309	30,165	26,658	53.1%	71.6%
Poland	359,952	129,861	121,841	51.6%	69.9%
Portugal	149,408	67,802	58,597	53.6%	84.6%
Romania	117,441	49,150	39,453	55.5%	75.4%
Russian Federation	940,103	180,330	257,350	41.2%	46.6%
Saudi Arabia	157,392	49,126	66,259	42.6%	73.3%
Serbia	57,701	25,065	22,165	53.1%	81.9%
Somalia	1,057	101	325	23.7%	40.3%
South Africa	141,129	45,024	55,139	45.0%	71.0%
Spain	801,513	291,516	310,020	48.5%	75.1%
Sri Lanka	43,124	4,210	6,172	40.6%	24.1%
Sweden	207,170	69,994	84,672	45.3%	74.7%
Switzerland	245,044	70,646	131,979	34.9%	82.7%
Syrian Arab Republic	8,132	2,525	3,694	40.6%	76.5%
Tunisia	62,780	22,157	18,328	54.7%	64.5%
Türkiye	440,360	183,318	208,001	46.8%	88.9%
United Arab Emirates	34,293	9,507	10,544	47.4%	58.5%
United Kingdom	1,282,607	389,777	490,191	44.3%	68.6%
United States	6,560,519	2,119,214	2,711,315	43.9%	73.6%
Zimbabwe	10,497	2,495	4,781	34.3%	69.3%

Table S8: Estimated slope (β_c) and coefficient of determination (R^2) for each country. Standard errors (SEs) and p-values are computed using a Newey-West estimator to account for potential autocorrelation.

Country	β_c	R^2	SE	p-value
Algeria	0.1537	0.9726	0.0082	< 0.0001
Egypt	0.1473	0.9938	0.0031	< 0.0001
Iraq	0.2613	0.9769	0.0099	< 0.0001
Jordan	0.1429	0.9933	0.0021	< 0.0001
Lebanon	0.1210	0.9829	0.0053	< 0.0001
Morocco	0.1063	0.9874	0.0032	< 0.0001
Saudi Arabia	0.1771	0.9800	0.0067	< 0.0001
Tunisia	0.1262	0.9253	0.0115	< 0.0001
United Arab Emirates	0.1569	0.9973	0.0015	< 0.0001
Australia	0.0572	0.9564	0.0040	< 0.0001
Canada	0.0502	0.9324	0.0045	< 0.0001
France	0.0418	0.9207	0.0039	< 0.0001
Germany	0.0519	0.9549	0.0036	< 0.0001
Italy	0.0576	0.9754	0.0030	< 0.0001
Japan	0.0192	0.9042	0.0022	< 0.0001
Spain	0.0696	0.9659	0.0041	< 0.0001
Sweden	0.0423	0.9521	0.0032	< 0.0001
United Kingdom	0.0473	0.9660	0.0029	< 0.0001
United States	0.0420	0.9678	0.0026	< 0.0001

Table S9: Estimated slope (γ_c) and coefficient of determination (R^2) for each country. Standard errors (SEs) and p-values are computed using a Newey-West estimator to account for potential autocorrelation.

Country	γ_c	R^2	SE	p-value
Algeria	0.0075	0.9598	0.0004	< 0.0001
Egypt	0.0086	0.9719	0.0003	< 0.0001
Iraq	0.0082	0.8495	0.0007	< 0.0001
Jordan	0.0110	0.9487	0.0006	< 0.0001
Lebanon	0.0075	0.9048	0.0007	< 0.0001
Morocco	0.0029	0.6891	0.0005	< 0.0001
Saudi Arabia	0.0106	0.9138	0.0010	< 0.0001
Tunisia	0.0139	0.9759	0.0006	< 0.0001
United Arab Emirates	0.0098	0.9700	0.0003	< 0.0001
Australia	0.0069	0.9833	0.0003	< 0.0001
Canada	0.0071	0.9951	0.0001	< 0.0001
France	0.0061	0.9716	0.0003	< 0.0001
Germany	0.0074	0.9861	0.0003	< 0.0001
Italy	0.0048	0.9604	0.0003	< 0.0001
Japan	0.0041	0.9940	0.0001	< 0.0001
Spain	0.0065	0.9849	0.0002	< 0.0001
Sweden	0.0060	0.9800	0.0002	< 0.0001
United Kingdom	0.0066	0.9970	0.0001	< 0.0001
United States	0.0072	0.9956	0.0001	< 0.0001

Table S10: Inter-publication interval thresholds (in years) for female and male authors when the survival probability threshold is set to 1%, 2%, and 5%.

	1%		2%		5%	
Country	Female	Male	Female	Male	Female	Male
Albania	8.18	9.88	6.92	7.09	4.67	4.33
Algeria	10.12	9.50	7.75	6.68	5.01	3.98
Australia	11.93	9.26	6.88	5.26	3.08	2.36
Austria	10.40	7.85	6.16	4.52	3.02	2.09
Bahrain	13.93	13.80	9.76	8.53	5.09	4.01
Belarus	11.52	11.27	8.46	7.45	4.70	3.83
Belgium	7.59	5.83	4.40	3.25	2.09	1.58
Bosnia and Herzegovina	10.01	10.01	7.01	6.93	4.05	4.01
Bulgaria	12.01	13.64	7.93	8.56	4.01	4.07
Canada	13.76	10.51	8.20	6.17	3.97	2.92
Cyprus	8.76	6.26	5.79	4.09	3.22	2.16
Czechia	8.01	7.92	5.16	4.68	2.84	2.25
Denmark	10.61	7.92	6.10	4.50	3.00	2.01
Egypt	13.75	14.01	8.68	8.67	4.61	4.21
Estonia	8.72	7.74	5.95	5.01	3.43	2.68
France	10.08	8.92	6.13	5.25	3.17	2.59
Germany	12.68	9.01	7.17	5.07	3.36	2.33
Ghana	10.47	9.01	6.89	6.01	4.01	3.17
Iceland	10.01	9.26	6.82	5.92	3.73	3.01
Iran, Islamic Republic of	8.18	6.92	6.01	4.72	3.57	2.46
Iraq	10.93	11.60	7.87	7.74	4.73	4.17
Ireland	15.65	11.93	9.01	6.67	4.01	2.91
Israel	12.01	9.18	7.36	5.47	3.81	2.67
Italy	9.18	7.51	5.17	4.22	2.45	2.00
Jamaica	20.03	20.10	12.60	13.35	6.19	6.59
Japan	16.58	10.25	10.28	6.01	4.52	2.50
Jordan	8.75	9.08	6.09	6.24	3.54	3.42
Kenya	10.06	10.60	7.25	7.01	4.15	4.00
Kuwait	12.04	11.35	8.81	7.50	4.89	4.01
Lebanon	11.93	11.01	7.09	6.84	3.61	3.17
Lithuania	6.67	7.09	4.92	4.98	3.00	2.77
Moldova, Republic of	10.09	10.43	7.01	7.01	4.01	4.01
Montenegro	10.52	10.01	7.01	6.84	4.01	3.59
Morocco	8.77	8.71	5.71	5.62	3.01	2.87
Netherlands	8.56	7.82	5.16	4.64	2.67	2.17
New Zealand	15.01	10.57	8.59	6.18	4.00	2.92
Nigeria	10.38	10.30	7.05	6.98	4.05	3.85
Norway	10.01	9.01	6.32	5.56	3.30	2.76
Oman	11.01	8.73	8.10	6.13	4.64	3.18
Philippines	14.36	13.10	9.87	8.53	5.62	4.42
Poland	9.01	8.39	5.67	5.11	3.01	2.83

Portugal	9.92	9.01	6.02	5.42	3.01	2.52
Romania	9.77	9.01	5.90	5.51	2.94	2.82
Russian Federation	10.01	11.30	6.47	6.92	3.34	3.25
Saudi Arabia	8.45	7.95	5.69	5.05	3.13	2.59
Serbia	9.51	9.84	5.84	6.01	3.00	3.01
South Africa	13.25	11.34	7.92	6.93	3.90	3.26
Spain	10.89	9.10	6.73	5.50	3.12	2.58
Sri Lanka	10.09	12.01	7.09	8.26	3.98	4.26
Sweden	10.01	7.76	6.05	4.64	3.06	2.18
Switzerland	11.31	8.79	6.68	5.01	3.21	2.34
Syrian Arab Republic	16.56	16.01	11.09	10.66	5.34	5.64
Tunisia	8.26	7.68	5.71	5.01	3.23	2.60
Türkiye	7.34	7.02	5.01	4.73	2.77	2.50
United Arab Emirates	10.75	8.01	6.92	5.13	3.67	2.58
United Kingdom	15.52	11.01	8.96	6.25	3.96	2.76
United States	14.27	11.84	8.51	7.01	4.01	3.09
Zimbabwe	12.01	10.76	8.11	7.44	4.84	4.22

Table S11: Spearman's rank correlation between cumulative productivity and two conventional career metrics, total productivity (TP) and publishing-career length (PCL).

	Female		Male	
Country	TP	PCL	TP	PCL
Albania	0.961	0.689	0.965	0.718
Algeria	0.975	0.636	0.973	0.698
Australia	0.957	0.677	0.937	0.662
Austria	0.951	0.672	0.924	0.657
Bahrain	0.986	0.504	0.969	0.533
Belarus	0.977	0.540	0.957	0.573
Belgium	0.908	0.648	0.882	0.619
Bosnia and Herzegovina	0.972	0.700	0.974	0.720
Bulgaria	0.976	0.677	0.971	0.663
Canada	0.958	0.644	0.934	0.639
Cyprus	0.970	0.700	0.947	0.730
Czechia	0.947	0.693	0.949	0.694
Denmark	0.957	0.699	0.933	0.677
Egypt	0.982	0.619	0.976	0.631
Estonia	0.957	0.723	0.944	0.687
France	0.949	0.688	0.932	0.670
Germany	0.957	0.643	0.924	0.639
Ghana	0.978	0.604	0.975	0.677
Iceland	0.957	0.698	0.952	0.719
Iran, Islamic Republic of	0.961	0.648	0.944	0.706
Iraq	0.981	0.539	0.979	0.571
Ireland	0.970	0.651	0.948	0.668
Israel	0.954	0.659	0.938	0.679
Italy	0.942	0.656	0.933	0.648
Jamaica	0.977	0.611	0.966	0.613
Japan	0.945	0.563	0.892	0.556
Jordan	0.973	0.566	0.963	0.673
Kenya	0.975	0.636	0.969	0.673
Kuwait	0.970	0.602	0.955	0.661
Lebanon	0.973	0.616	0.966	0.672
Lithuania	0.954	0.754	0.945	0.732
Moldova, Republic of	0.978	0.614	0.977	0.638
Montenegro	0.978	0.714	0.978	0.710
Morocco	0.969	0.616	0.971	0.678
Netherlands	0.945	0.693	0.921	0.669
New Zealand	0.967	0.674	0.948	0.680
Nigeria	0.975	0.552	0.973	0.608
Norway	0.960	0.715	0.944	0.694
Oman	0.973	0.485	0.949	0.652
Philippines	0.982	0.473	0.979	0.504

Country	TP	PCL	TP	PCL
Poland	0.966	0.723	0.957	0.703
Portugal	0.965	0.705	0.951	0.687
Romania	0.965	0.710	0.960	0.716
Russian Federation	0.981	0.632	0.976	0.618
Saudi Arabia	0.979	0.567	0.966	0.662
Serbia	0.974	0.738	0.973	0.729
South Africa	0.971	0.676	0.960	0.675
Spain	0.954	0.666	0.939	0.661
Sri Lanka	0.971	0.598	0.977	0.607
Sweden	0.943	0.673	0.921	0.638
Switzerland	0.958	0.671	0.925	0.657
Syrian Arab Republic	0.986	0.439	0.978	0.484
Tunisia	0.954	0.666	0.945	0.698
Türkiye	0.961	0.721	0.949	0.725
United Arab Emirates	0.980	0.546	0.967	0.691
United Kingdom	0.964	0.646	0.938	0.639
United States	0.959	0.624	0.937	0.614
Zimbabwe	0.970	0.624	0.961	0.654

Table S12: Proportion of active authors in 2023 with a publication gap experience.

Country	Female (%)	Male (%)
Albania	7.87	9.97
Algeria	5.52	10.69
Australia	17.58	25.50
Austria	13.60	23.52
Bahrain	3.14	6.88
Belarus	5.03	9.77
Belgium	22.79	32.30
Bosnia and Herzegovina	7.97	12.04
Bulgaria	10.76	11.76
Canada	12.75	20.47
Cyprus	11.96	20.70
Czechia	17.25	23.76
Denmark	15.32	25.46
Egypt	6.09	8.51
Estonia	14.11	19.89
France	16.07	21.93
Germany	11.66	22.43
Ghana	4.41	8.64
Iceland	17.09	21.55
Iran, Islamic Republic of	9.08	19.17
Iraq	3.90	5.51
Ireland	12.83	19.97
Israel	11.66	19.21
Italy	22.12	28.37
Jamaica	6.31	7.62
Japan	13.04	29.59
Jordan	5.29	10.83
Kenya	6.83	9.34
Kuwait	6.35	11.52
Lebanon	7.12	12.55
Lithuania	16.29	19.46
Moldova, Republic of	6.63	8.67
Montenegro	9.26	14.43
Morocco	8.63	14.20
Netherlands	17.58	25.78
New Zealand	12.36	20.90
Nigeria	4.81	6.36
Norway	14.67	21.29
Oman	3.89	12.22
Philippines	2.85	4.08
Poland	15.56	21.78
Portugal	20.87	25.46
Romania	12.24	15.57

Country	Female (%)	Male (%)
Russian Federation	7.34	8.40
Saudi Arabia	5.34	11.49
Serbia	16.08	17.08
South Africa	9.72	13.53
Spain	17.11	24.76
Sri Lanka	6.59	5.60
Sweden	20.20	29.48
Switzerland	13.23	22.33
Syrian Arab Republic	2.88	4.98
Tunisia	12.61	21.59
Türkiye	13.89	21.08
United Arab Emirates	5.10	13.53
United Kingdom	12.43	21.75
United States	12.48	19.49
Zimbabwe	5.55	8.66

Table S13: Proportion of returning authors within newly active authors in 2023.

Country	Female (%)	Male (%)
Albania	17.56	18.82
Algeria	7.35	11.07
Australia	16.87	24.22
Austria	12.51	25.09
Bahrain	1.66	6.82
Belarus	5.94	10.71
Belgium	24.22	34.90
Bosnia and Herzegovina	8.45	13.62
Bulgaria	9.76	10.83
Canada	12.86	22.26
Cyprus	13.08	20.73
Czechia	19.34	23.93
Denmark	14.37	25.84
Egypt	4.42	7.18
Estonia	15.22	21.03
France	17.21	22.53
Germany	11.27	23.46
Ghana	4.29	8.65
Iceland	18.56	19.40
Iran, Islamic Republic of	11.85	21.75
Iraq	4.20	6.28
Ireland	11.94	19.56
Israel	11.75	18.59
Italy	20.46	25.42
Jamaica	13.64	4.17
Japan	13.79	34.83
Jordan	3.66	9.64
Kenya	6.23	9.35
Kuwait	7.65	14.32
Lebanon	5.03	7.28
Lithuania	14.71	21.62
Moldova, Republic of	6.99	9.66
Montenegro	8.97	9.76
Morocco	5.56	9.28
Netherlands	16.32	25.55
New Zealand	13.87	20.33
Nigeria	5.22	8.06
Norway	13.33	20.88
Oman	3.51	10.84
Philippines	2.18	3.51
Poland	16.10	21.68
Portugal	22.42	27.22
Romania	12.72	15.65

Country	Female (%)	Male (%)
Russian Federation	7.54	7.62
Saudi Arabia	3.99	7.71
Serbia	16.89	20.07
South Africa	8.56	14.20
Spain	17.27	23.33
Sri Lanka	4.88	6.86
Sweden	22.98	34.45
Switzerland	11.88	22.69
Syrian Arab Republic	3.54	3.02
Tunisia	13.08	18.49
Türkiye	12.42	20.08
United Arab Emirates	3.80	8.95
United Kingdom	12.00	23.20
United States	11.94	20.65
Zimbabwe	7.76	10.42

Table S14: Researcher inflow and gender gap in cumulative productivity in 2023.

Country	Researcher inflow	Gender gap in cumulative productivity
Albania	12.26%	−31.89%
Algeria	10.30%	−46.19%
Australia	7.68%	−35.75%
Austria	9.53%	−47.00%
Bahrain	13.86%	−46.77%
Belarus	6.63%	−35.28%
Belgium	11.46%	−40.18%
Bosnia and Herzegovina	8.16%	−32.45%
Bulgaria	7.11%	−6.30%
Canada	7.45%	−40.34%
Cyprus	11.02%	−50.00%
Czechia	8.44%	−43.01%
Denmark	8.79%	−47.70%
Egypt	10.70%	−23.70%
Estonia	8.27%	−44.60%
France	9.11%	−34.78%
Germany	8.49%	−51.63%
Ghana	14.30%	−42.75%
Iceland	8.11%	−36.59%
Iran, Islamic Republic of	9.16%	−54.02%
Iraq	15.33%	−26.50%
Ireland	7.35%	−37.79%
Israel	8.85%	−40.69%
Italy	9.98%	−29.86%
Jamaica	4.95%	−14.81%
Japan	6.64%	−56.26%
Jordan	15.56%	−47.23%
Kenya	11.89%	−30.67%
Kuwait	10.43%	−39.39%
Lebanon	10.99%	−42.69%
Lithuania	9.99%	−32.43%
Moldova, Republic of	11.59%	−20.74%
Montenegro	7.54%	−35.03%
Morocco	14.08%	−38.63%
Netherlands	9.78%	−40.39%
New Zealand	6.90%	−42.72%
Nigeria	12.72%	−22.80%
Norway	8.49%	−38.37%
Oman	13.51%	−59.85%
Philippines	17.70%	−29.79%
Poland	8.14%	−30.25%
Portugal	8.44%	−23.57%

Country	Researcher inflow	Gender gap in cumulative productivity
Romania	9.83%	−17.79%
Russian Federation	7.80%	−15.78%
Saudi Arabia	17.63%	−48.85%
Serbia	6.98%	−7.94%
South Africa	8.70%	−31.44%
Spain	8.66%	−34.10%
Sri Lanka	10.46%	1.84%
Sweden	7.09%	−39.90%
Switzerland	9.09%	−46.99%
Syrian Arab Republic	15.17%	−33.67%
Tunisia	11.48%	−46.08%
Türkiye	12.07%	−35.74%
United Arab Emirates	15.68%	−59.48%
United Kingdom	7.18%	−40.19%
United States	7.44%	−37.03%
Zimbabwe	10.73%	−29.31%

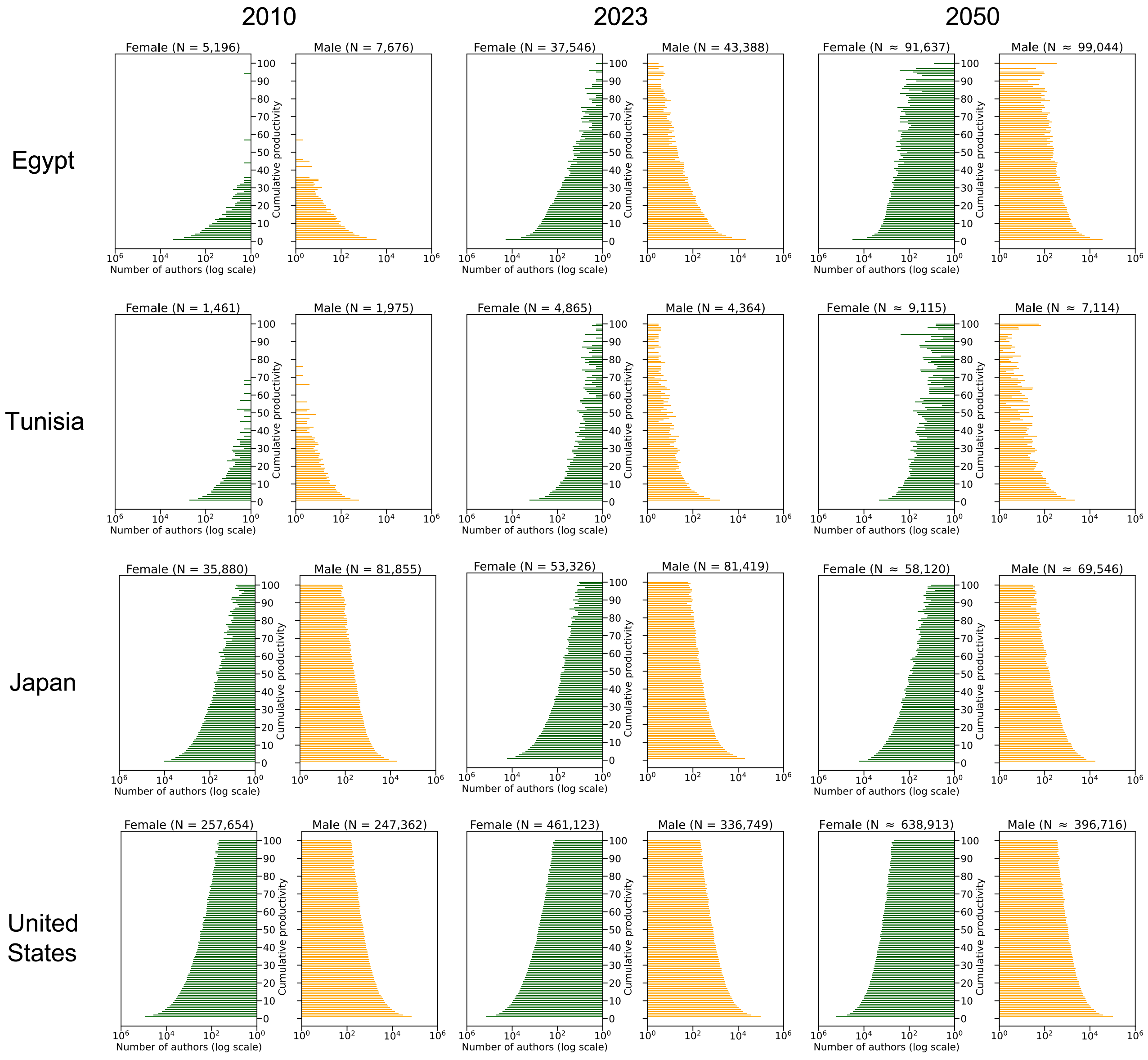

Figure S1: Researcher population pyramids in 2010, 2023, and 2050 for Egypt, Tunisia, Japan, and the United States in Health Sciences. The number of active authors for each gender is displayed on a logarithmic horizontal axis. Total active author counts (N) are provided for each panel. The 2050 pyramids and their corresponding counts ($\approx$) are projections based on 2023 trends.

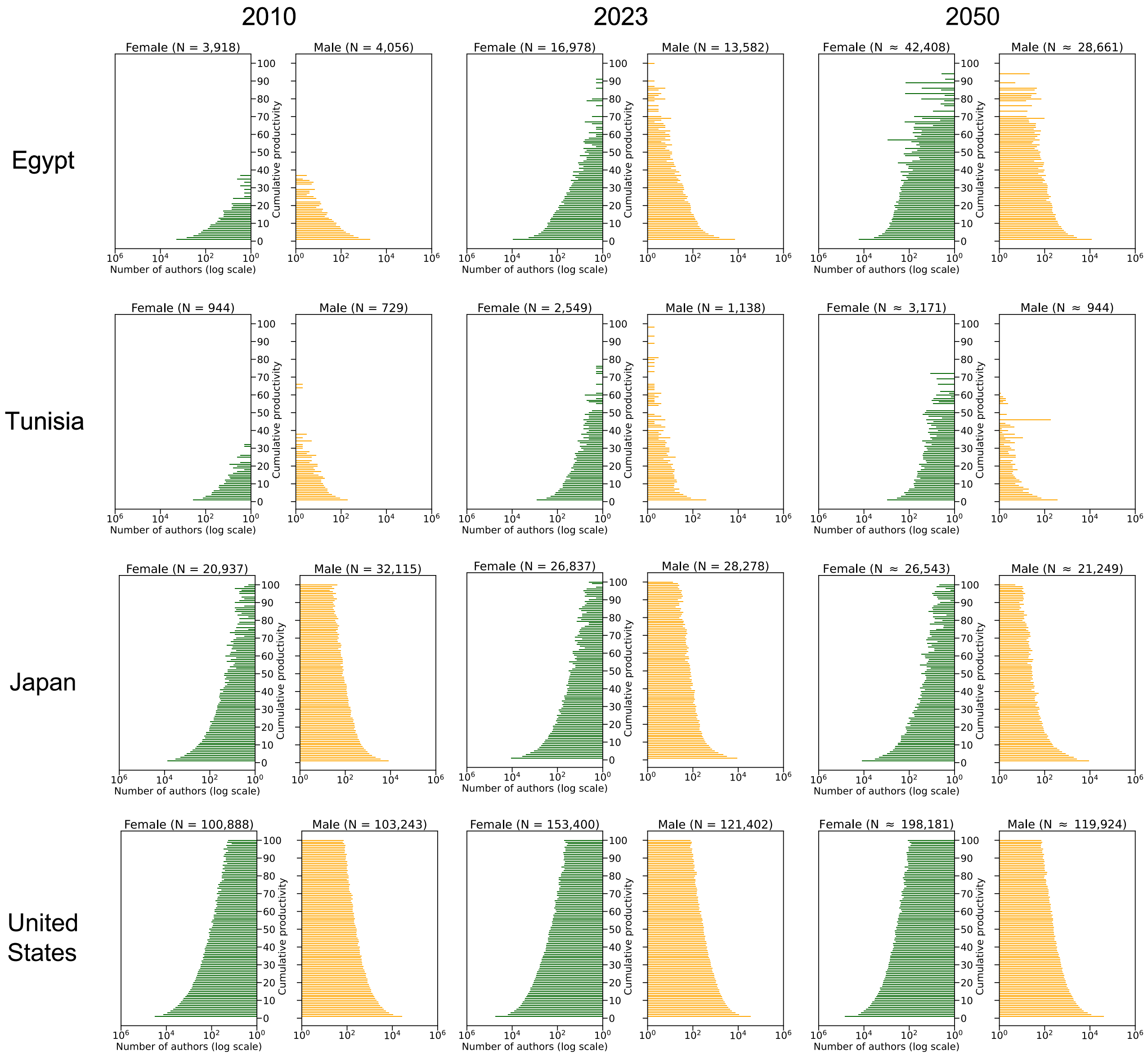

Figure S2: Researcher population pyramids in 2010, 2023, and 2050 for Egypt, Tunisia, Japan, and the United States in Life Sciences. The number of active authors for each gender is displayed on a logarithmic horizontal axis. Total active author counts (N) are provided for each panel. The 2050 pyramids and their corresponding counts ($\approx$) are projections based on 2023 trends.

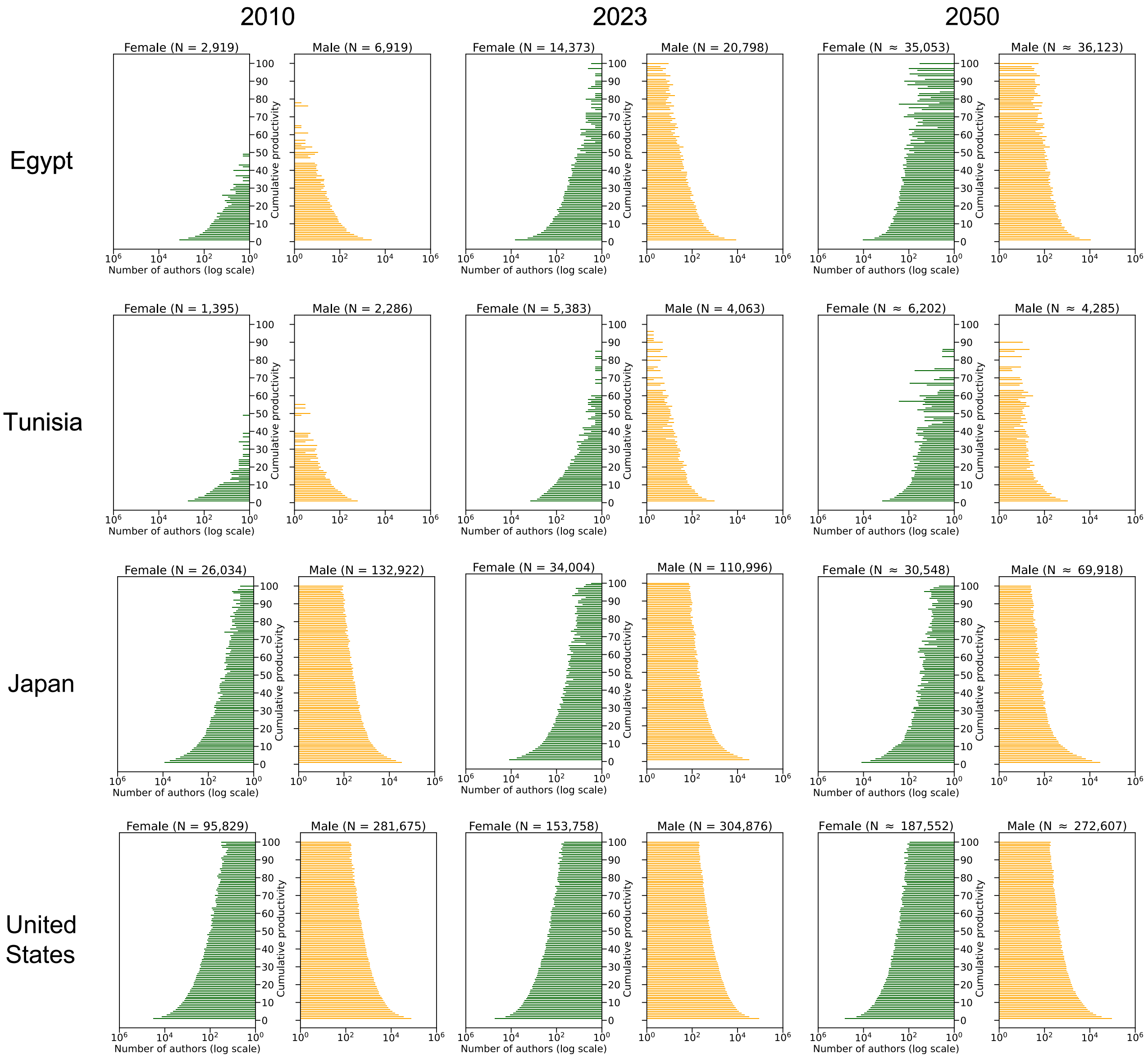

Figure S3: Researcher population pyramids in 2010, 2023, and 2050 for Egypt, Tunisia, Japan, and the United States in Physical Sciences. The number of active authors for each gender is displayed on a logarithmic horizontal axis. Total active author counts (N) are provided for each panel. The 2050 pyramids and their corresponding counts ($\approx$) are projections based on 2023 trends.

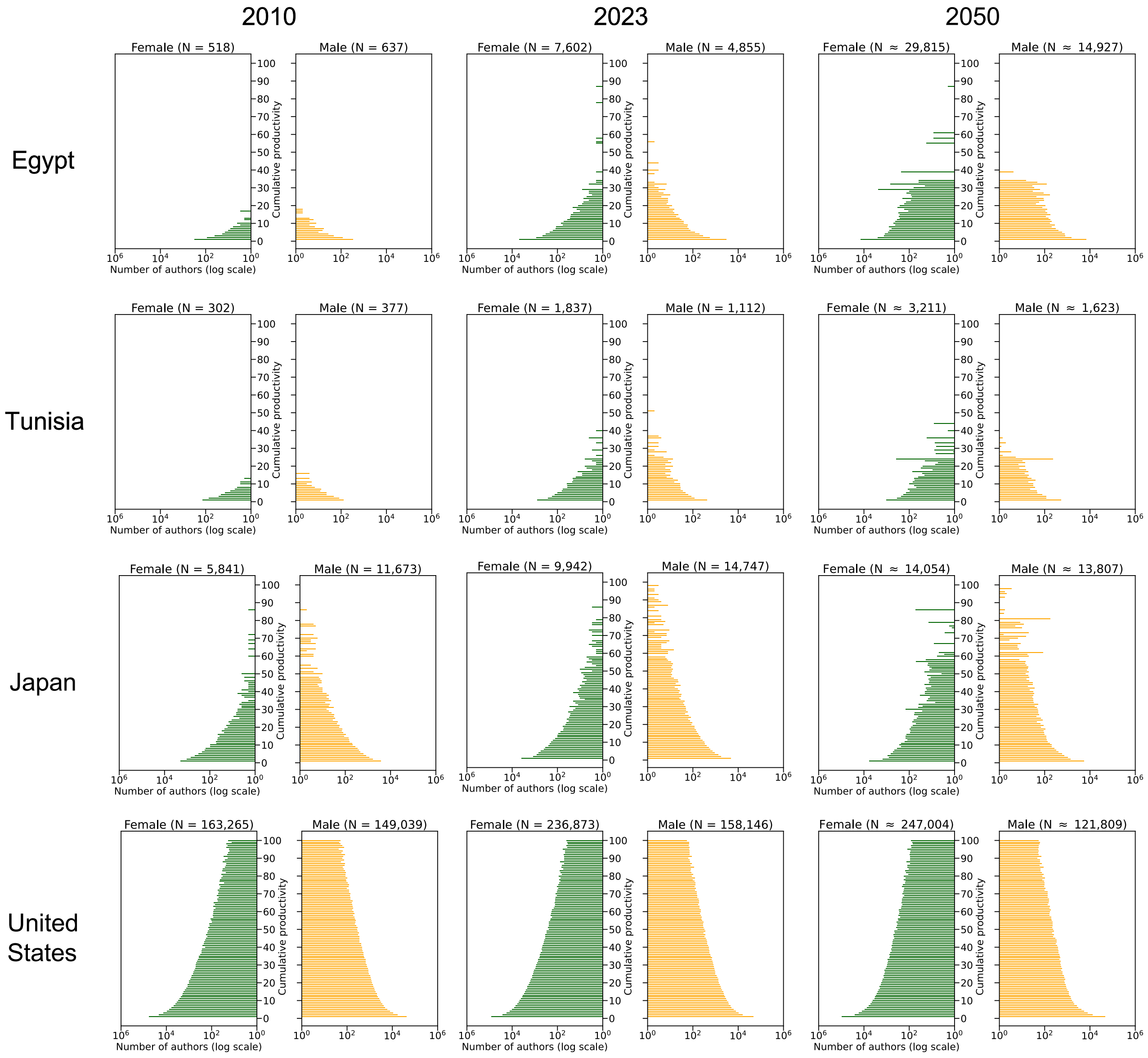

Figure S4: Researcher population pyramids in 2010, 2023, and 2050 for Egypt, Tunisia, Japan, and the United States in Social Sciences. The number of active authors for each gender is displayed on a logarithmic horizontal axis. Total active author counts (N) are provided for each panel. The 2050 pyramids and their corresponding counts ($\approx$) are projections based on 2023 trends.
